# Antioxidant Activity and Antifungal Activity of Chitosan Derivatives with Propane Sulfonate Groups

**DOI:** 10.3390/polym10040395

**Published:** 2018-04-03

**Authors:** Fang Luan, Lijie Wei, Jingjing Zhang, Yingqi Mi, Fang Dong, Qing Li, Zhanyong Guo

**Affiliations:** 1 Key Laboratory of Coastal Biology and Bioresource Utilization, Yantai Institute of Coastal Zone Research, Chinese Academy of Sciences, Yantai 264003, China; fluan@yic.ac.cn (F.L.); ljwei@yic.ac.cn (L.W.); jingjingzhang@yic.ac.cn (J.Z.); yqmi@yic.ac.cn (Y.M.); qli@yic.ac.cn (Q.L.); 2 University of Chinese Academy of Sciences, Beijing 100049, China

**Keywords:** chitosan derivative, sulfonated chitosan, antioxidant ability, antifungal activity

## Abstract

We successfully synthesized the water-soluble chitosan derivatives propane sulfonated chitosan (PSCS) and dipropane sulfonated chitosan (DPSCS) in this paper. These derivatives were characterized by FTIR, ^1^H NMR, and ^13^C NMR. Moreover, the antioxidant activity of the chitosan derivatives was evaluated by free radical scavenging ability (against DPPH-radical, hydroxyl-radical, and superoxide-radical) and ferric reducing power. Meanwhile, inhibitory effects against two fungi were also tested. Our results suggested antioxidant abilities and antifungal properties were in order of DPSCS > PSCS > CS, which were consistent with the number of propane sulfonated groups. The scavenging activity of DPSCS against superoxide-radical and DPPH-radical were 94.1% and 100% at 1.6 mg/mL, respectively. The inhibitory indices of DPSCS against *P. asparagi* and *F. oxysporum* were up to 82.2% and 94% at 1.0 mg/mL, respectively. Obviously, the number of propane sulfonated groups of chitosan derivatives not only contributes to antioxidant activity, but also to antifungal activity. Therefore, DPSCS with more propane sulfonated groups endowed with antioxidant and antifungal activity that can be used as a candidate material in the food and pharmaceutical industries.

## 1. Introduction

Chitosan (CS) is a natural polymer obtained from chitin and it is the only natural cationic polysaccharide known. This unique property makes it extensively used in foods, cosmetics, biomedicals, and agriculture [1,2,3]. Compared with chitosan, chitosan derivatives have been found important and widely applied in various fields. Water-insoluble chitosan derivatives have been widely used in environmentally friendly adsorbent material, drug delivery, treatment of ophthalmic diseases, and so on [4,5,6,7]. Moreover, water-soluble chitosan derivatives have a wide range of applications, including antisclerotic, antibacterial, antioxidant, wound dressing, and enzyme inhibition activities, etc. [8,9,10,11].

Sulfonation can enhance the water-solubility of derivatives due to the addition of sulfonic acid groups [12], and polysaccharides without sulfate groups exhibit biological activities after sulfation, e.g., antiviral, hemagglutination inhibition, adsorption of metal ions, and controlling the activity of growth factors in cells [13,14,15]. Thus, attention has been attracted to the sulfonation of chitosan, and a series of sulfonated chitosans, including *O*-sulfated chitosan, *N*-sulfated chitosan, and *N*, *O*-sulfated chitosan, were synthesized. As expected, these derivatives have higher antitumor, anticoagulant, and antibacterial activity [16,17,18,19,20]. To our knowledge, there are few papers about chitosan derivatives with sulfonated side chains. Pendent sulfonated chitosan could be prepared by modifying chitosan using 1,3-propane sultone and it could be used as biopolymer membrane and water-born antimicrobial additive [21,22,23].

The antioxidant property has attracted attention in food and pharmaceutical industries due to the ability to prevent chain initiation, bind transition metal ions, decompose peroxides, and so on. In the present study, we firstly synthesized 6-amino-6-deoxychitosan (NCS). Then, CS and NCS were reacted with 1,3-propane sultone to generate propane sulfonated chitosan (PSCS) and dipropane sulfonated chitosan (DPSCS), both of which were water-soluble. Polysaccharide derivatives can be characterized via various analysis methods including elemental analysis, FT-IR, ^1^H NMR, and ^13^C NMR. Then, we evaluated its antioxidant ability and antifungal activity. In our report, scavenging activities (hydroxyl-radical, superoxide-radical and against DPPH-radical) and reducing power tests were employed to test the antioxidant activity. Meanwhile, inhibitory effects against fungi were tested using *Phomopsis asparagi* (*P. asparagi*) and *Fusarium oxysporum* (*F. oxysporum*). The relationship between the structure and the antioxidant and antifungal activities of chitosan was discussed.

## 2. Materials and Methods

### 2.1. Materials

Chitosan was supplied by Zhejiang Golden-Shell Pharmaceutical Co. Ltd. (Yuhuan, China), and the molecular weight was 50 kDa and its degree of deacetylation (DD) was 92%. The elemental analyses (C, H, S, and N) were performed on a Vario Micro Elemental Analyzer (Elementar, Germany). The other reagents such as *N*,*N*-Dimethylformamide (DMF), *N*-bromobutanimide (NBS), triphenylphosphine, *N*-Methyl pyrrolidone (NMP), etc., were supplied by Sinopharm Chemical Reagent Co., Ltd. (Shanghai, China).

### 2.2. Analytical Methods

FT-IR spectra were measured on a Jasco-4100 Fourier Transform Infrared Spectrometer (JASCO Co., Ltd. Shanghai, China) with KBr disks. The elemental analyses (C, H, and N) were performed on a Vario Micro Elemental Analyzer (Elementar, Germany). The UV–Vis absorbance was measured with a T6 New Century UV spectrometer (P General Co., Ltd., Beijing, China). ^13^C Nuclear Magnetic Resonance (^13^C NMR) spectra were measured with a Bruker AVANCE III Spectroscopy (Bruker Tech. and Serv. Co., Ltd. Beijing, China) The degrees of substitution (DS) were calculated on the basis of the percentages of carbon and nitrogen.

### 2.3. Synthesis

**Scheme 1:**
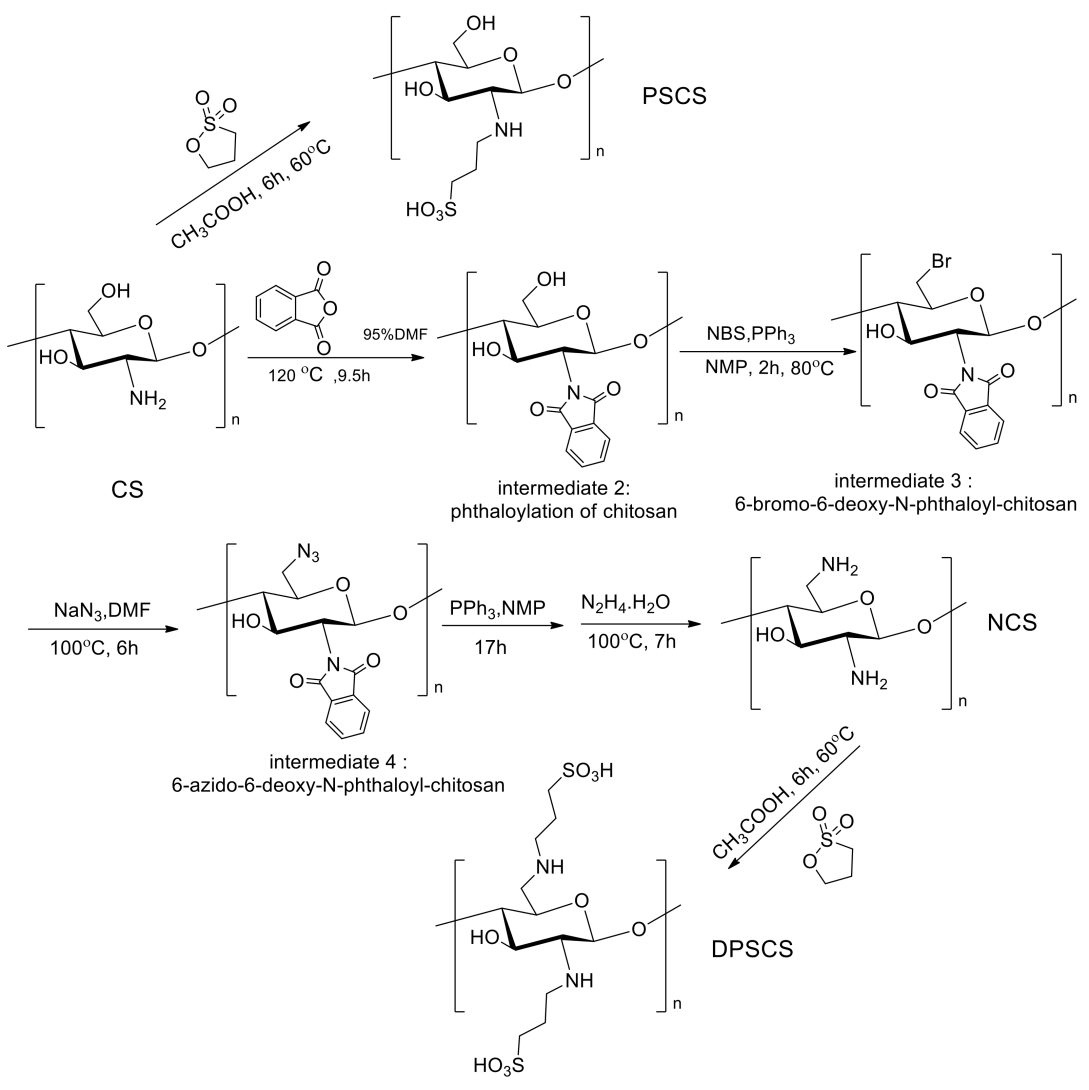
Synthetic route for the preparation of PSCS and DPSCS.

As shown in Scheme 1, NCS was prepared according to the literature [24]. PSCS and PDSCS was prepared by the method described by Sun with minor modifications [23].

#### 2.3.1. 6-Deoxy-*N*-phthaloyl-chitosan (Intermediate **2**)

15.1 g of phthalic anhydride was dissolved in 300 mL DMF containing 5% (*v*/*v*) water. Then, 5.1 g of CS was added to the solution, and the mixture was heated at 120 °C. After 9.5 h of reaction, the resulting mixture was cooled to room temperature and gradually poured into ice water. The precipitate was collected on a filter, washed two times with distilled water and ethanol, and dried at 60 °C. The product was obtained as a flesh-colored powdery material.

#### 2.3.2. Preparation of 6-Bromo-6-deoxy-*N*-phthaloyl-chitosan (Intermediate **3**)

3.5 g phthaloylated chitosan (**2**) was dissolved in 330 mL DMF, then 21.4 g NBS and 31.4 g triphenylphosphine were added slowly in an ice bath. The mixture was stirred at 80 °C for 2 h. The reaction mixture was poured into ethanol, and the resulting precipitate was collected by filtration.

#### 2.3.3. Preparation of 6-Azido-6-deoxy-*N*-phthaloyl-chitosan (Intermediate **4**)

A mixture of 1.6 g 6-bromo-6-deoxy-*N*-phthaloyl-chitosan (**3**) and 50 mL DMF was treated with sodium azide (2.0 g) at 100 °C for 7 h. The resulting mixture was cooled to room temperature and stirred overnight. The solution was then poured into 400 mL ice water. The precipitate was collected, washed with distilled water, ethanol, and then dried at 60 °C to give the product.

#### 2.3.4. Preparation of NCS

0.95 g 6-azido-6-deoxy-*N*-phthaloyl-chitosan (**4**) was suspended in 100 mL DMF. Then, 2.67 g triphenylphosphine was added. After 20 h of reaction at room temperature, 95 mL hydrazine monohydrate was then added to the mixture and the mixture was heated to 100 °C. After 8 h of reaction, the resulting solid was washed with 200 mL of ethanol and dried to give 6-amino-6-deoxychitosan.

#### 2.3.5. Preparation of Dipropane Sulfonated Chitosan (DPSCS)

0.25 g of NCS was completely dissolved in 30 mL of 2% acetic acid solution. Afterwards, 3.0 mL of 1,3-propane sultone was added into solution, and the solution was stirred at 60 °C for 6 h. The resulting mixture was dialyzed for 2 days and lyophilized.

#### 2.3.6. Preparation of Propane Sulfonated Chitosan (PSCS)

The synthesis method of PSCS is similar to that of DPSCS, except the amount of 1,3-propane sultone was halved.

### 2.4. Investigation of the Antioxidant Ability

#### 2.4.1. Hydroxyl-Radical Scavenging Ability Assay

The test of the hydroxyl-radical scavenging ability was carried out according to Hu’s methods [25]. The reaction mixture, with a total volume of 4.5 mL, containing testing samples (CS, PSCS, and DPSCS), were incubated with EDTA-Fe^2+^ (220 μmol/L), safranine T (0.23 μmol/L), and H_2_O_2_ (60 μmol/L) in potassium phosphate buffer (150 mM, pH 7.4) for 30 min at 37 °C. The absorbance of the mixture was measured at 520 nm. Three replicates for each sample were tested and the scavenging effect was calculated according to the following Equation (1):

(1) $$\mathrm{Scavenging}\;\mathrm{effect}\;\left(\%\right)=\frac{A_{\mathrm{sample}\;520\;\mathrm{nm}}\;-\;A_{\mathrm{blank}\;520\;\mathrm{nm}}\;}{A_{\mathrm{control}\;520\;\mathrm{nm}}-A_{\mathrm{blank}\;520\;\mathrm{nm}}}\times 100$$

where *A*_sample 520 nm_ is the absorbance of the sample at 520 nm; *A*_control 520 nm_ is the absorbance of the control (distilled water instead of H_2_O_2_) at 520 nm; and *A*_blank 520 nm_ is the absorbance of the blank (distilled water instead samples) at 520 nm.

#### 2.4.2. Superoxide-Radical Scavenging Ability Assay

The superoxide-radical scavenging ability was assessed following the model of Wei’s methods with minor modification [26]. Involving testing samples (CS, PSCS, and DPSCS), 30 μmol phenazine mothosulfate (PMS), 338 μmol nicotinamide adenine dinucleotide reduced (NADH), and 72 μmol nitro blue tetrazolium (NBT) in Tris-HCl buffer (16 mM, pH 8.0), the reaction mixture was incubated at 25 °C for 5 min. The absorbance of the mixture was measured at 560 nm. Three replicates for each sample were tested and the scavenging effect was calculated according to the following Equation (2):

(2) $$\mathrm{Scavenging}\;\mathrm{effect}\;\left(\%\right)=\left[1-\frac{A_{\mathrm{sample}\;560\;\mathrm{nm}}\;-\;A_{\mathrm{control}\;560\;\mathrm{nm}}\;}{A_{\mathrm{blank}\;560\;\mathrm{nm}}}\right]\times 100$$

where *A*_sample 560 nm_ is the absorbance of the sample at 560 nm; *A*_control 560 nm_ is the absorbance of the control (distilled water instead of NADH) at 560 nm; and *A*_blank 560 nm_ is the absorbance of the blank (distilled water instead samples) at 560 nm.

#### 2.4.3. DPPH-Radical Scavenging Ability Assay

The DPPH scavenging properties of the products were evaluated by the following method [27]. Different concentrations of samples (CS, PSCS, and DPSCS) and 2 mL of DPPH ethanol solution (180 μmol/L) were incubated for 30 min at room temperature. Then, the absorbance of the remaining DPPH radical was measured at 517 nm against a blank. Three replicates for each sample were tested and the scavenging effect was calculated according to the following Equation (3):

(3) $$\mathrm{Scavenging}\;\mathrm{effect}\;\left(\%\right)=\left[1-\frac{A_{\mathrm{sample}\;517\;\mathrm{nm}}\;-\;A_{\mathrm{control}\;517\;\mathrm{nm}}\;}{A_{\mathrm{blank}\;517\;\mathrm{nm}}}\right]\times 100$$

where *A*_sample 517 nm_ is the absorbance of the sample (with DPPH) at 517 nm; *A*_control 517 nm_ is the absorbance of the control (without DPPH) at 517 nm; and *A*_blank 517 nm_ is the absorbance of the blank (without samples) at 517 nm.

#### 2.4.4. Reducing Power Assay

The reducing power was determined according to the method of Zhong [28]. 1.5 mL of testing sample was mixed with 1.5 mL of 1% potassium ferricyanide, and the mixture was incubated at 50 °C for 20 min. Thereafter, 1.5 mL of 10% trichloroacetic acid was added. The upper layer (2.0 mL) was mixed with 2.0 mL of water and 0.2 mL of 0.1% ferric chloride, and the absorbance was measured at 700 nm. A higher absorbance indicates a stronger reducing power [29].

### 2.5. Evaluation of Antifungal Activity In Vitro

Antifungal assays were performed by following the plate growth rate method described by Li et al. [30]. Briefly, each sample solution was added to Fungi Medium to give final concentrations of 0.1, 0.5, and 1.0 mg/mL. Triadimefon solution with the same concentration was used as a positive control. After the mixture was cooled in the plate, 5.0 mm diameter of fungi mycelium was transferred to the test plate and incubated at 27 °C for 3 days. When fungi mycelium in the control plate (without samples) reached the edges, the antifungal index was calculated as follows Equation (4):

(4) $$\mathrm{Antifungal}\;\mathrm{index}\;\left(\%\right)=\left[1-\frac{D_{a}\;}{D_{b}}\right]\times 100$$

where *D*_a_ is the diameter of growth zone in test plate and *D*_b_ is the diameter of growth zone in control plate. Each experiment was performed in three replicates.

## 3. Results and Discussion

### 3.1. Chitosan Derivatives Preparation and Characterization

#### 3.1.1. Synthesis of Chitosan Derivatives

The 6-amino-6-deoxy chitosan was prepared in five steps, namely the phthaloylation, bromination, azidation, reduction, and deprotection of the phthaloyl group. Raw chitosan was insoluble in water and the resulting product, 6-amino-6-deoxy chitosan, also had poor water-solubility. Then, we synthesized the water-soluble chitosan derivatives PSCS and DPSCS by sulfonylation. Their solubilities in water were up to 50 mg/mL, and good water solubility permits wider use. The degree of substitution (DS) is shown in Table 1, as determined by the C/N (intermediate **2**, intermediate **3**, intermediate **4** and NCS) or S/N (PSCS and DPSCS) ratio from the elemental analysis.

#### 3.1.2. FT-IR Analysis

**Figure 1:**
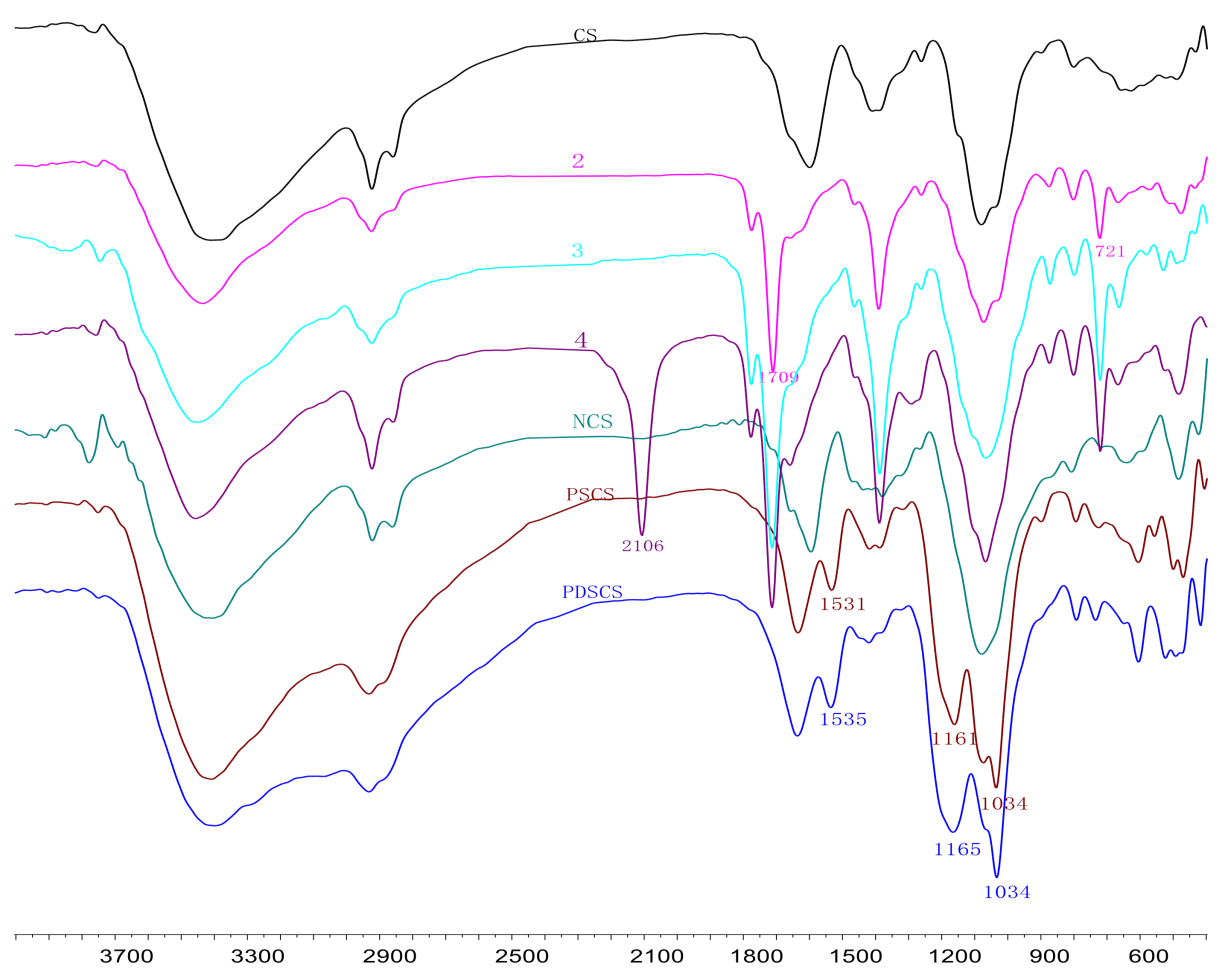
IR spectra of the chitosan (CS), phthaloylation of chitosan (intermediate **2**), 6-bromo-6-deoxy-*N*-phthaloyl-chitosan (intermediate **3**), 6-azido-6-deoxy-*N*-phthaloyl-chitosan (intermediate **4**), 6-amino-6-deoxychitosan (NCS), propane sulfonated chitosan (PSCS), and dipropane sulfonated chitosan (DPSCS).

FT-IR spectroscopy has been demonstrated to be a powerful tool for studying the physicochemical properties of polysaccharides. The syntheses were confirmed by the FT-IR showed in Figure 1.

For CS, intense adsorption at around 3400 cm^−1^ corresponding to the stretching vibration of –OH and –NH_2_. The band at around 1600 cm^−1^ was assigned to bending vibration of –NH_2_. The twin strong absorptions at around 1080 and 1030 cm^−1^ were assigned to the C–O stretching vibration of the second hydroxyl group and primary hydroxyl group, respectively [31]. The spectra of *N*-phthaloyl-chitosan showed two strong characteristic absorptions at 1774 cm^−1^ and 1709 cm^−1^ which were related to stretching vibration of C=O. The peak at 721 cm^−1^ was due to the bending vibration of C–H in the aromatic ring. Although there was no obvious peak at around 530 cm^−1^ in spectra of compound **3**, the C–O stretching band at 1036 cm^−1^ corresponding to the primary hydroxyl group disappeared, which verified bromination of the C_6_–OH. For compound **4**, a new peak at around 2106 cm^−1^ appeared and can be attributed to the stretching vibration of the azide group. It also confirmed the successful synthesis of the previous step.

For NCS, the peak at 2106 cm^−1^ disappeared. At the same time, absorptions at 1774 cm^−1^ and 1709 cm^−1^ also disappeared, which indicated that the azide and *N*–phthaloyl groups were removed. Meanwhile, the peak at around 1600 cm^−1^ was assigned to the –NH_2_ group. Compared with CS, the C–O stretching band at 1030 cm^−1^ related to the primary hydroxyl group disappeared, which also confirmed the successful synthesis.

Finally, for PSCS/DPSCS, the intense adsorption at around 1034 and 1165 cm^−1^, corresponding to the S=O stretching vibration from the sulfonic acid group, peak at around 1535 cm^−1^ was attributed to the C–N–C bending vibration of the PSCS/DPSCS branch, which confirmed the synthesis of desired compound.

#### 3.1.3. NMR Analysis

**Figure 2:**
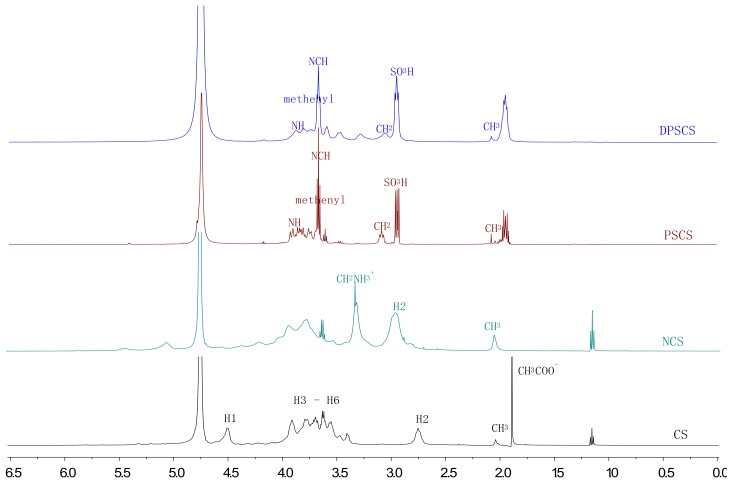
^1^H NMR spectra of chitosan derivatives.

**Figure 3:**
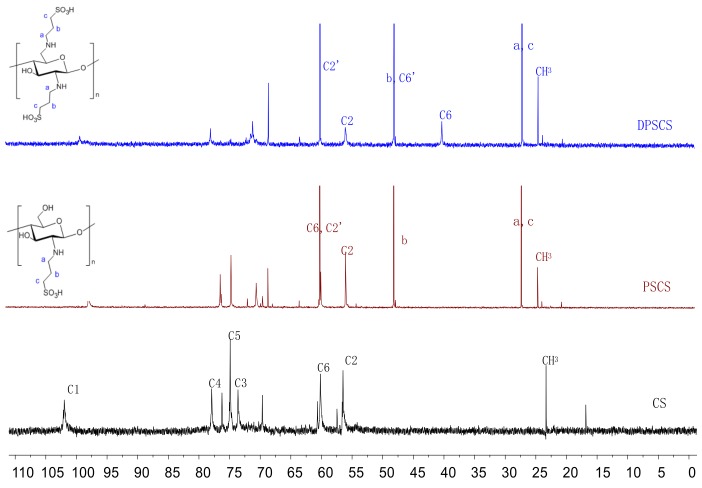
^13^C NMR spectra of chitosan derivatives.

PSCS and DPSCS are water-soluble. Due to the solubility of products, we only got ^1^H NMR of the chitosan and 6-amino-6-deoxychitosan which were dissolved with D_2_O–CH_3_COOH and D_2_O–DCl, respectively. The ^1^H NMR and ^13^C NMR spectra of chitosan derivatives are shown in Figure 2 and Figure 3. In the ^1^H NMR spectrum of CS, the peaks at 2.0 ppm and 1.9 ppm corresponded to methyl (CH_3_ group) and CH_3_COO^−^ groups, respectively. The peak at around 2.8 ppm was attributed to H2, peaks at 3.9 ppm to 3.4 ppm were attributed to H3–H6, peak at 4.5 ppm was attributed to H1. The signal at 4.8 ppm was related to solvent D_2_O and the signals at 3.6 ppm and 1.2 ppm were related to residue of ethanol. In the ^1^H NMR spectrum of NCS, a signal at 4.8 ppm was related to solvent D_2_O, the signals at 3.0 was corresponding H2, and the new peak at 3.3 ppm was attributed to –CH_2_NH_3_^+^ at C6, which suggested the amino groups were incorporated into the C6 position of chitosan. For PSCS, the signal at 3.1 ppm and 2.9 ppm were attributed to the –CH_2_ and –SO_3_H groups in 1,3-propane sultone, and the peak at 3.7 ppm was attributed to –NCH substituted at C2, which suggested amino groups had been substituted. The peak at 3.1 ppm caused by incomplete substitution at C2. It indicated that free amino groups at C2 were partly substituted. This spectrum is also in agreement with the results reported by Sun [23]. The diffused peaks nearby to 2.0 ppm is due the overlapping of the signals comes out from the overlapping of –OH and –NH groups in the form of –CH_2_N [21]. Similarly, in the spectrum of DPSCS, the signal at 3.1 ppm and 2.9 ppm were attributed to the –CH_2_ and –SO_3_H groups in 1,3-propane sultone, and the peak at 3.7 was attributed to –NCH substituted. Meanwhile, the signals at 3.1 and 3.3 ppm were still observed. It meant some amino at C2 and C6 did not react.

In the ^13^C NMR spectrum of CS, the peak at around 102 ppm was attributed to C1, peaks at 78 ppm to 73 ppm were attributed to C3, C4 and C5, peaks at 60 ppm and 56 ppm was attributed to C6 and C2, respectively. The signal at 24 ppm was attributed to methyl (CH_3_ group). In the ^13^C NMR spectrum of PSCS, the signals at 27 ppm and 48 ppm were attributed to the carbons in 1,3-propane sultone [12]. The C2–NH_2_ involved in the reaction was labeled as C2′ and C2–NH_2_, which did not participate in the reaction, was labeled as C2. The signal at 60 ppm was assigned to the replaced C2′; in addition, the signal at 56 ppm was attributed to incomplete substitution at C2. It indicated that free amino groups at C2 were partly substituted. For DPSCS, the signals at 27 ppm and 48 ppm were attributed to the carbons in 1,3-propane sultone. Similarly, the C6–NH_2_ involved in the reaction was labeled as C6′ and C6–NH_2_, which did not participate in the reaction, was labeled as C6. And the signals at 48 ppm and 60 ppm were assigned to the replaced C6′ and C2′, respectively. In addition, the signals at 40 and 56 ppm was still observed. It meant some of amino at C2 and C6 did not react. Based on these results, it is concluded that the 1,3 propane sultone reacted with the amino groups of the chitosan polymer.

### 3.2. Antioxidant Activities

#### 3.2.1. Hydroxyl-Radical Scavenging Ability Assay

**Figure 4:**
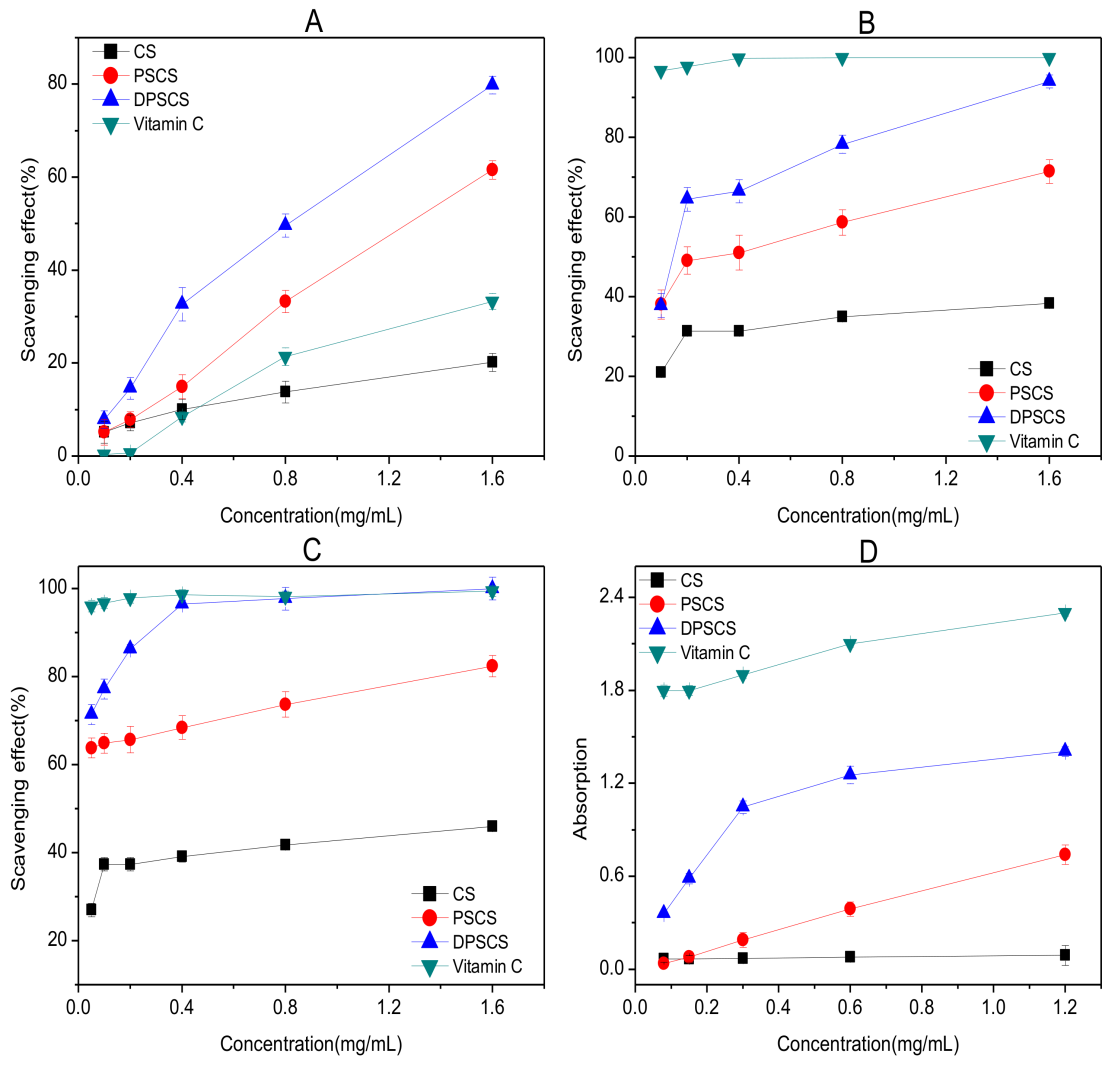
Antioxidant effect of samples: (**A**) Hydroxyl-radical scavenging ability; (**B**) Superoxide-radical scavenging ability; (**C**) DPPH-radical scavenging ability; (**D**) Reducing power.

Hydroxyl-radical is the most reactive free radical and can be formed from superoxide anion and hydrogen peroxide which can react with living cells and induce severe damage [3]. Figure 4A shows the curve chart of the hydroxyl-radical scavenging ability of CS, PSCS, and DPSCS at various concentrations. Compared with Vitamin C as a control standard, the scavenging effect increased as the concentration of the polymer samples increased. The scavenging ability against hydroxyl-radical was in order of DPSCS > PSCS > CS in the tested concentration range. Results showed that DPSCS and PSCS exhibited remarkable improvement on hydroxyl-radical scavenging activity; the scavenging effects were 79.9% and 61.6% at 1.6 mg/mL, respectively. By contrast, the scavenging effects of Vitamin C and CS were only 30.3% and 20.2%, respectively.

The scavenging activity may be related to active hydrogen in chitosan derivatives. And active hydrogen in the polysaccharide unit can react with OH by the typical H abstraction reaction. The propane sulfonate groups grafted on CS can donate active hydrogen. The more active hydrogen, the higher scavenging ability. Thus, DPSCS had the highest scavenging activity. We conclude that active hydrogen could be a positive factor that affects the scavenging activity against hydroxyl-radicals.

#### 3.2.2. Superoxide-Radical Scavenging Ability Assay

Superoxide anions are one of the precursors of the singlet oxygen and hydroxyl radicals and they can damage cells and DNA, leading to various diseases [32]. Superoxide scavenging activity was determined in the NBT assay. As shown in Figure 4B, the superoxide-radical scavenging ability of the obtained derivatives was similar to the scavenging properties against hydroxyl-radicals. Also, the scavenging ability of polymer samples increases with concentration. In addition, Vitamin C used as a positive control, could scavenge superoxide-radical totally at 0.4 mg/mL. DPSCS also had the excellently scavenging activity, reaching 94.1% at 1.6 mg/mL. The maximum of 71.5% and 38.4% inhibition was observed at the concentration of 1.6 mg/mL of PSCS and CS.

As we known, the scavenging effect is related to the number of active hydrogens in the molecule. As discussed in the hydroxyl-radical assay, PSCS and DPSCS can more easily release hydrogen that reacts with superoxide anions. Therefore, an increased number of propane sulfonated groups in DPSCS promotes the scavenging effect. The results, again, suggest that DPSCS can be considered as efficient antioxidant polymers and propane sulfonated groups play a role in their free radical scavenging ability.

#### 3.2.3. DPPH-Radical Scavenging Ability Assay

DPPH is a useful reagent for investigating the free radical-scavenging activity of compounds. This method is based on the reduction of alcoholic DPPH solution in the presence of antioxidant into non-radical DPPH-H, and the reduction in color is monitored over time [33]. The scavenging property of CS, PSCS, and DPSCS against DPPH-radical at various concentrations is shown in Figure 4C. According to the graph, we could conclude the results as follows: firstly, the scavenging effect increased as the concentration of the polymer samples increased to a certain extent, and then levelled off even with further increase in the concentration. Secondly, Vitamin C had the best scavenging activity and the scavenging ability against DPPH-radical was in order of DPSCS > PSCS > CS in the tested concentration range. Thirdly, the DPSCS, PSCS, and CS showed antioxidant activities of 100%, 82.4%, and 46% at 1.6 mg/mL, respectively. Furthermore, the scavenging activity of DPSCS was close to Vitamin C at 0.8 mg/mL.

The scavenging activity may be related to DPPH-radicals reacting with active hydrogen in chitosan derivatives to form a stable macromolecule. The principle has been discussed in Section 3.2.1 and Section 3.2.2. Therefore, DPSCS exhibited the highest DPPH-radical scavenging ability. In other words, propane sulfonated groups are an important factor that influences the scavenging activity against DPPH-radicals.

#### 3.2.4. Reducing Power Assay

The ferric ion reducing power assay tests reducing power based on an electron transfer reaction. In the assay, the presence of reductants (antioxidants) results in the reduction of the ferric ion/ferricyanide complex to the ferrous form, with a characteristic formation of Perl’s Prussian blue, which can be measured spectrophotometrically [34]. From Figure 4D, we can clearly see that the reducing power of polymer samples correlated well with the increasing concentration. Vitamin C, used as a positive control, had a reducing power of 2.3 at 1.2 mg/mL. It showed that the reducing power of samples were significantly lower than that of Vitamin C. DPSCS exhibited stronger reducing power than PSCS and CS, and they showed reducing power of 1.41, 0.74, and 0.1 at 1.2 mg/mL, respectively.

Accordingly, the reducing capacity of a compound may serve as a significant indicator of its potential antioxidant activity [35]. Our results on the reducing power suggest that propane sulfonated groups likely contribute significantly to the reducing power.

### 3.3. Antifungal Activity

**Figure 5:**
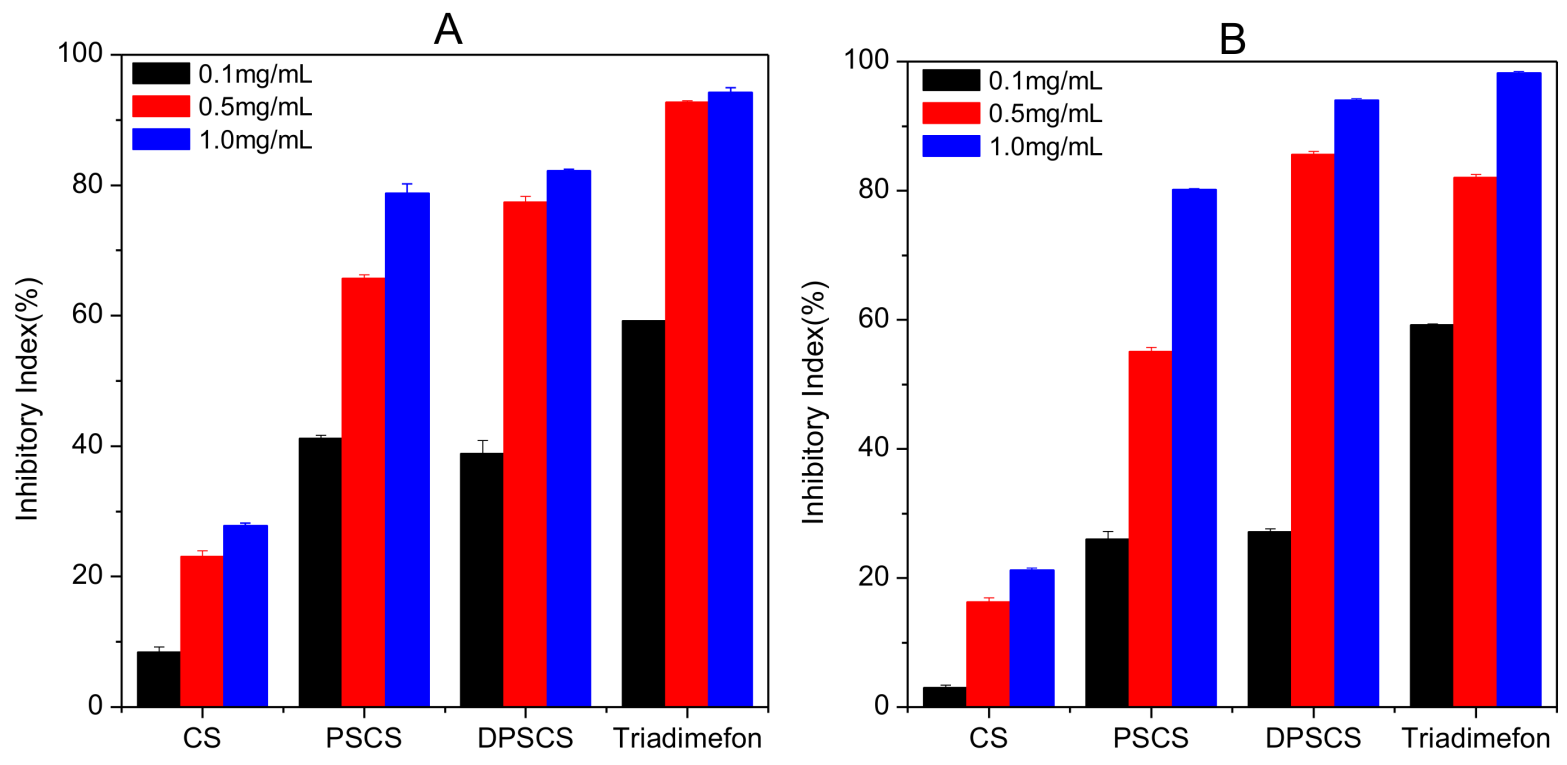
Antifungal activity against *P. asparagi* (**A**) and *F. oxysporum* (**B**) at 0.1, 0.5, and 1.0 mg/mL.

The action of fungi causes the most economically important seed or plant diseases. Control of these plant-threatening fungi can benefit vegetable and fruit production. We tested the antifungal activity of CS, PSCS, and DPSCS against common plant-threatening fungi *P. asparagi* and *F. oxysporum*. The antifungal activity assayed herein is shown in Figure 5. As illustrated in the graph, the antifungal activities of all the samples exhibited an upward trend with the increase of sample concentration and the strongest antifungal activity was observed at 1.0 mg/mL. Meanwhile, inhibitory index was in order of DPSCS > PSCS > CS. Details are as follows.

As shown in Figure 5A, CS slightly inhibited the growth of the tested fungi and the inhibitory indices was 27.8% at 1.0 mg/mL. Compared to CS, inhibitory indices of PSCS, DPSCS, and triadimefon were up to 78.8%, 82.2%, and 94.2% at 1.0 mg/mL, respectively. That is to say, PSCS and DPSCS possess powerful antifungal activity against *P. asparagi* and propane sulfonated groups play a major role in the antifungal activity.

As shown in Figure 5B, CS also slightly inhibited the growth of the tested fungi and the inhibitory index was 21.2% at 1.0 mg/mL. Compared to CS, the inhibitory indices of PSCS and DPSCS were 80.2% and 94.0% at 1.0 mg/mL, respectively. Meanwhile, the inhibitory index of triadimefon (the positive control) was 98.2%. Besides, the antifungal activity of DPSCS was close to triadimefon. In other words, both PSCS and DPSCS inhibited *F. oxysporum* and propane sulfonated groups could be a positive factor that affects the antifungal activity.

Based on the results mentioned above, propane sulfonated groups are thought to be the functional antifungal groups. As a result, DPSCS with double propane sulfonated groups should have better antifungal activity than PSCS with single propane sulfonated groups and CS.

## 4. Conclusions

In summary, a series of derivatives of chitosan with single or double propane sulfonate groups were synthesized successfully. In addition, the antioxidant activity and inhibitory effects of PSCS and DPSCS were determined. Our results suggested that PSCS and DPSCS had higher antioxidant activities. The same order was observed for the antifungal activities of the derivatives. The antioxidant abilities and antifungal properties were in order of DPSCS > PSCS > CS. It is reasonable to presume that propane sulfonated groups are an important factor that influences both the antioxidant and antifungal activity. Therefore, DPSCS is endowed with antioxidant activity and can be used as a candidate material in the food and pharmaceutical industries.

## Figures and Tables

**Table 1:** The elemental analyses, yields, and the degrees of substitution of samples.

Name	N%	C%	H%	S%	DS	Yield
CS	8.49	44.72	6.88	*-*	*-*	*-*
intermediate **2**	4.68	52.92	4.45	*-*	DS_phthal__oyl_: 0.92	83%
intermediate **3**	4.24	47.78	3.91	*-*	DS_bromo_: 0.89	47%
intermediate **4**	15.99	51.66	4.15	*-*	DS_azido_: 0.88	98%
NCS	15.01	42.54	7.83	*-*	DS_amino_: 0.81	41%
PSCS	4.76	35.70	6.38	7.14	DS_sulfonate_: 0.66	45%
DPSCS	3.79	32.61	5.81	10.4	DS_sulfonate_: 1.2	57%
